# Use of extracorporeal endotoxin elimination therapy for septic shock

**DOI:** 10.1186/cc12004

**Published:** 2013-03-19

**Authors:** BA Adamik, JS Smiechowicz, SZ Zielinski, AK Kübler

**Affiliations:** 1 Medical University, Wroclaw, Poland

## Introduction

Endotoxin, a component of the outer membrane of Gram-negative bacteria, is considered an important factor in pathogenesis of septic shock [1]. The aim of our study was to determine whether endotoxin elimination treatment added to the standard treatment would improve organ function in patients with septic shock.

## Methods

Adult patients with septic shock who required renal replacement therapy (RRT), with a confirmed endotoxemia, and suspected Gram-negative infection were consecutively added to the study within the first 24 hours after diagnosis. All patients received full standard treatment for septic shock. Endotoxin elimination was performed using the membrane oXiris (Gambro, Sweden), a medical device for continued RRT with the unique feature of endotoxin adsorbtion. An endotoxin activity assay was used to monitor endotoxin elimination therapy at baseline (T0), 3 hours (T1), 12 hours (T2), 24 hours (T3), 48 hours (T4), and 72 hours (T5). Our key indicators were the improvement in hemodynamics and organ function, and decrease of endotoxin activity (EA) in blood. Continuous variables are presented as mean values with standard deviations.

## Results

High EA level at baseline (0.74 ± 0.14 endotoxin activity units (EAU)) significantly decreased during RRT with oXiris membrane to 0.46 ± 0.02 (T1), 0.34 ± 0.01 (T2), 0.4 ± 0.02 (T3), 0.46 ± 0.04 (T4), 0.35 ± 0.07 (T5) EAU (*P <*0.05). MAP increased from baseline 72 ± 14 to 81 ± 18, 76 ± 6, 77 ± 7, 83 ± 13, 87 ± 10 mmHg (*P <*0.05), and the mean norepinephrine use decreased from 0.23 ± 0.04 to 0.19 ± 0.02, 0.11 ± 0.01, 0.09 ± 0.01, 0.04 ± 0.01, 0.0 μg/kg/minute (*P <*0.05) at T0, T1, T2, T3, T4, T5, respectively. The SOFA score had decreased from 14 ± 4 to 12 ± 2, 9 ± 3, 7 ± 3 points (*P <*0.05), and the procalcitonin level declined from 107 ± 123 to 45 ± 41, 29 ± 30, 17 ± 157 ± 1 ng/ml (*P <*0.05) at T0, T3, T4, T5.

## Conclusion

RRT with oXiris membrane resulted in the effective elimination of endotoxins from the blood. The therapy was associated with an increase in blood pressure, a reduction of vasopressor requirements, and an improvement of organ function. The application of the endotoxin activity assay was useful for bedside monitoring of endotoxemia in ICU patients.
